# Conrad and Becker’s “10 Criteria” Fall Short of Addressing Conflicts of Interest in Chemical Safety Studies

**DOI:** 10.1289/ehp.1104385

**Published:** 2011-12-01

**Authors:** Patrice Sutton, Tracey J. Woodruff, Sarah Vogel, Lisa A. Bero

**Affiliations:** Program on Reproductive Health and the Environment, University of California, San Francisco, Oakland, California, E-mail: suttonp@obgyn.ucsf.edu; Johnson Family Foundation, New York, New York; Institute of Health Policy Studies, University of California, San Francisco, San Francisco, California

In their review “Enhancing Credibility of Chemical Safety Studies: Emerging Consensus on Key Assessment Criteria”, Conrad and Becker (2011) proposed the application of 10 criteria to individual studies as a means of assessing a study’s “credibility.” They characterized the 10 criteria as an “emerging consensus” and “encouraging convergence” that can solve “this problem of legitimacy in chemical evaluation … regardless of its funding source.” Conrad and Becker explicitly excluded funding by industry as a criterion for evaluating studies, labeling funding-based criteria as “unscientific”; they also dismissed outright consideration of developing potentially unbiased, third-party mechanisms for chemical safety testing as “too costly and complicated.” We agree with Conrad and Becker that there is an urgent unmet need to address the problem of conflict of interest in the science that underlies the regulation of chemicals used in commerce. However, Conrad and Becker’s 10 criteria fall short of what is needed in several critical ways.

Conrad and Becker (2011) mischaracterized the problem as one of “perception” and “public confidence” when there is empirical evidence of bias related to funding source. Chemical manufacturing represented a 700 billion dollar industry in the United States in 2007 (U.S. Census Bureau 2007); thus, the chemical industry is not a disinterested party in the science that informs chemical safety. In the clinical sciences, a robust association has been documented between funding from an industry that has a financial interest in the result, investigator conflicts of interest, and biased outcomes in human health research. For example, speaking on the issue of scientific integrity, the current Deputy Editor (West) of *JAMA* recently observed that “the biggest threat to integrity [is] financial conflict of interest” (Rennie 2010). Specific examples can be found in research related to the health effects of tobacco (Barnes and Bero 1997, 1998) and the safety and efficacy of pharmaceuticals (Bero et al. 2007; Lexchin et al. 2003) and medical procedures (Popelut et al. 2010; Shah et al. 2005). These and other empirical findings (Bero 1999) demonstrate that an industry’s financial stake is unlikely to be mitigated by applying a 10-point “credibility score” such as the one proposed by Conrad and Becker.

Conrad and Becker (2011) presented no evidence to support the capacity of a “credibility score” to reduce industry funding bias. As noted in the 2009 Institute of Medicine (IOM) report on *Conflicts of Interest in Medical Research, Education and Practice* (Institute of Medicine 2009), studies on the effects of industry relationships in nonclinical research, as well as studies of the consequences of conflict of interest policies in nonclinical research, are needed to establish an evidence base for future policy. The IOM report made no recommendations for managing conflicts of interest in animal or laboratory research because of the lack of empirical data. This data gap should be filled with credible, empirically based research such as what has been used to evaluate these questions in the clinical sciences. We (L.A.B. and T.W.) are currently engaged in such research.

Individually and together, Conrad and Becker’s 10 criteria do not have the capacity to mitigate funding bias. Their criteria are a mixture of minimal measures of scientific integrity (full disclosure, independence of principal investigators, prohibiting ghost writing, peer review); strongly contested judgments related to the superiority of study designs [Good Laboratory Practices (GLP) versus non-GLP studies]; and practices that lack the capacity to reduce bias unless they are wedded to mechanisms for transparent, unconflicted enforcement and accountability (registries and organizational existence of an external review policy). Although their scoring system admirably requires disclosure, their criteria standards explicitly ignored what is disclosed as a potential source of bias when evaluating the quality of a study. Moreover, disclosure does not itself eliminate bias (Bero 1999).

There is an inherent conflict in the source documents from which Conrad and Becker (2011) drew their “consensus” criteria, with two of them funded directly by industry sources (Henry and Conrad 2008; Rowe et al. 2009). Henry and Conrad (2008) were funded by the American Chemistry Council, and Rowe et. al.’s paper on financial conflicts and scientific integrity in nutrition research (Rowe et al. 2009) was supported by the International Life Sciences Institute (ILSI) North America. ILSI’s programs are supported primarily through ILSI North America industry membership; the nutrition paper was also specifically supported by Cadbury Adams USA LLC, Coca-Cola Company, ConAgra Foods Inc., General Mills, Kraft Foods, Mars Snackfoods US LLC, PepsiCo Inc., Proctor and Gamble, Sara Lee, and Tate and Lyle.

It is significant to note that the three source documents not directly funded by industry [Bipartisan Policy Center 2009; Brockway and Furcht 2006; International Agency for Research on Cancer (IARC) 2008] (although the coauthors may include industry representatives) explicitly recognized the problem of funding as a source of bias. For example, while recognizing that studies should not be excluded *a priori* due to funding source, the Bipartisan Policy Center report states that

Agencies and scientific advisory committees should consider sources of funding and any conflicts of interest as they review the reasons why a study may have been undertaken, the way a study was framed and carried out, and how the study results have been interpreted and discussed. (Bipartisan Policy Center 2009)

IARC (2008) precludes research funding from commissioning parties that “develop activities or sustain principles that are contrary to IARC missions, e.g., the tobacco industry”; and FASEB (the Federation of American Societies for Experimental Biology) lists as a guiding principle that “investigators shall regard all significant financial interests in research regarding human subjects as potentially problematic and thus requiring close scrutiny”(Brockway and Furcht 2006). A closer read of the source documents is inconsistent with Conrad and Becker’s attribution of an “emerging consensus” on this issue.

We are not suggesting that industry-funded science should *a priori* be excluded from the evidence informing chemical policy. We are proposing that based on the evidence from the clinical sciences, an important pathway to addressing funding bias is through the application of systematic and transparent methodologies to vet the science, including explicit recognition of the potential bias introduced by funding source (Guyatt et al. 2008; Higgins and Green 2006; Woodruff et al. 2011) Importantly, although essential to addressing bias, systematic and transparent methods still do not guarantee that the influence of industry funding will be eliminated (Rennie 2010; Roseman et al. 2011)

Ultimately, the only legitimate pathway to achieve public confidence about chemical safety is to institute and sustain a proactive, robust regulatory system that is responsive to the needs of individuals, workers, and communities whose health is impacted by exposure to chemicals (Landrigan and Goldman 2011; Vogel and Roberts 2011). Advances in chemical policy must be inextricably linked to the development and support for independent mechanisms to generate the science, systematic and transparent reviews of the evidence, and unceasing regulatory and public vigilance about the influence of funding source on the science that informs environmental health policy.
